# Treasurer’s Report for Financial Year (FY) 2019

**DOI:** 10.1093/molbev/msab012

**Published:** 2021-05-03

**Authors:** Jeffrey L. Thorne, John M. Archibald

The society’s total assets as of December 31, 2019, are as follows:

**Table:** 

Cash account	$1,573,218.77
**Total CD**	$2,084,692.50
**Prepaid expenses**	$147,522.77
**Accounts receivable**	$84,640.70
**Total assets**	$3,890,074.74

This report covers 7 months from June 1, 2019 to December 31, 2019. The society continues to be financially healthy. For this 7-month period, the revenue collected by the society was $426,068.94 with the main sources of revenue being our two journals, *Molecular Biology and Evolution* (*MBE*) and *Genome Biology and Evolution* (*GBE*). These funds facilitated our support of the annual SMBE meeting that was held in Manchester, England, in 2019. SMBE provided a total of 43 graduate and postdoctoral travel awards as well as registration-only awards for an additional 46 young scientists. The society also supported the Walter Fitch Prize Symposium (a cash prize for the winner and travel funds for all eight finalists); the Undergraduate Diversity Mentoring Program; the travel and accommodation of Council members; and travel incentives for associate editors, senior editors, and editors-in-chief of our journals. The society subsidized on-site childcare at the Manchester meeting and provided additional financial support to families attending the meeting via its Carer award program. For the fifth consecutive year, we were able to fund 100% of applications for the Carer award. In addition, SMBE again subsidized prizes that recognized achievements of scientists at all career stages. Further investments in the field of molecular evolution were made by SMBE through sponsorship of diverse satellite (regional) meetings.

So that the fiscal and calendar years will coincide after FY2019, the 2019 financial year was only 7 months. This fiscal year modification was made by the SMBE Council at the 2019 annual meeting. The rationale was that the two SMBE journals are the main sources of revenue for the society and the publication contracts for these journals specify that the annual amounts payable to SMBE are determined on a calendar year basis. For fiscal year 2020 and beyond, Treasurer’s reports will therefore directly reflect the annual journal revenues of the associated year.

The change of fiscal year has two main impacts on FY2019. First, because most SMBE expenses each year are attributable to the annual SMBE meeting while the period covered here only corresponds to 7 months of journal revenues, the disparity between revenues and expenses is bigger for fiscal year 2019 than it would have been if the period had represented 12 months. Second, Treasurer’s reports prior to FY2019 were based on estimates of the journal revenues for the last 5 months of each fiscal year (i.e., January through May) because those 5 months are at the beginning of the calendar year, but journal revenue amounts were determined following the end of the calendar year. Because *MBE* and *GBE* revenues for the last 5 months of FY2018 were overestimated in the FY2018 report, the adjustment for those estimation errors is made here and results in a lower total revenue for FY2019. Because of the change in fiscal year, such adjustments will not be needed in future reports.

**Table:** 

Revenues	
Membership dues	$13 454.79
*MBE* FY2019 revenue	$224 627.00
*GBE* FY2019 revenue	$136,654.00
Unrestricted donation	$150.00
Nei Lecture Fund donation	$0.00
Interest income	$51,183.15
***Total revenues***	$***426,068.94***
**Expenses**	
Senior editor travel	$13,325.43
Associate editors’ travel	$113,822.25
Past/present editors-in-chief and journal staff travel	$15,971.29
Council travel	$18,808.17
Fitch Symposium travel	$18,198.70
Undergraduate diversity travel	$14,407.47
Young investigator travel	$89,400.90
***Total travel***	**$*283,934.21***
Miscellaneous prize & career awards	$23,509.57
Carer awards	$48,825.16
***Total prize & awards***	**$*72,334.73***
Operating expenses	$45,306.17
Bank charges	$479.82
Legal fees	$1,652.89
***Total operations***	**$*47,438.88***
*MBE* editing	$57,118.36
*MBE* highlight honoraria	$3,592.00
*GBE* editing	$64,166.70
*GBE* highlight honoraria	$10,619.00
***Total editorial***	**$*135,496.06***
Annual SMBE meeting	$298,999.42
Other meeting/workshop sponsorships	$67,574.00
***Total meetings***	**$*366,573.42***
***Total expenses***	$***905,777.30***
***Change in net assets***	***-***$***479,708.36***

